# Implementing One-at-a-Time Therapy in community addiction and mental health centres: a retrospective exploration of the implementation process and initial outcomes

**DOI:** 10.1186/s12913-023-09923-5

**Published:** 2023-09-12

**Authors:** Laura M. Harris-Lane, Natalie R. Keeler-Villa, Alexa Bol, Katie Burke, AnnMarie Churchill, Peter Cornish, Sarah F. Fitzgerald, Bernard Goguen, Kristina Gordon, Alexia Jaouich, Rino Lang, Mylène Michaud, Kaitlyn N. Mahon, Joshua A. Rash

**Affiliations:** 1 Department of Psychology, Memorial University of Newfoundland, 230 Elizabeth Ave, St. John’s, NL A1B 3X9 Canada; 2 Stepped Care Solutions, Mount Pearl, Canada; 3 Department of Health, Addiction & Mental Health Services, Government of New Brunswick, Fredericton, New Brunswick Canada; 4 Counseling and Psychological Services, University of California, Berkeley, Berkeley, USA; 5 Addiction & Mental Health Services, Horizon Health Network, Fredericton, New Brunswick Canada; 6 Mental Health & Addictions Services, Vitalité Health Network, Bathurst, New Brunswick Canada

**Keywords:** One-at-a-Time Therapy, Single session therapy, Stepped Care 2.0, Implementation science, System change, Mental health, Addiction

## Abstract

**Background:**

The Department of Health of the Government of New Brunswick and Regional Health Authorities elected to implement Stepped Care 2.0 (SC2.0) in 2021, and began with One-at-a-Time (OAAT) therapy in Community Addiction and Mental Health Centres (CAMHCs) to facilitate rapid access to addiction and mental healthcare. This study: 1) explicated the process of implementing OAAT therapy as it aligned to evidence-based implementation frameworks and strategies; 2) assessed readiness for change among providers during the implementation; and 3) evaluated initial client and system outcomes.

**Methods:**

The process of implementing OAAT therapy within CAMHCs was documented and retrospectively aligned with the Active Implementation Frameworks-Stages of Implementation, Consolidated Framework for Implementation Research, and incorporated strategies endorsed by the Expert Recommendations for Implementing Change. Providers working in CAMHCs completed online asynchronous courses in OAAT therapy and SC2.0, and were recruited to participate in research on perceptions of organizational readiness. Initial outcomes of the implementation were evaluated through client satisfaction surveys administered in CAMHCs and system performance indicators.

**Results:**

Aligning with implementation stages, key strategies included: 1) continuously monitoring readiness and soliciting stakeholder feedback for iterative improvement; 2) building a representative implementation team with engaged leaders; 3) creating a comprehensive implementation plan on staff training, communication, and system changes; and 4) supporting sustainability. Providers who participated in research (*N* = 170, ~ 50% response rate) agreed that their organization was ready for implementation, and that OAAT therapy delivered within a SC2.0 framework was acceptable, appropriate, and feasible. More than 3,600 OAAT therapy sessions were delivered during the initial implementation stage, and waitlists were reduced by 64.1%. The majority of clients who completed surveys (*N* = 1240, ~ 35% response rate) reported that their OAAT therapy session was helpful, with a minority reporting that additional intervention was needed.

**Conclusions:**

Thoughtful planning and execution, aligned with evidence-based implementation frameworks and strategies, played an important role in this provincial change initiative. Implementation steps outlined can help inform others looking to enact large-scale change.

**Supplementary Information:**

The online version contains supplementary material available at 10.1186/s12913-023-09923-5.

## Background

Stepped Care 2.0 (SC2.0) is a transformative model for the delivery of mental health, substance use, and addiction resources [1]. Clients have rapid access to care within the SC2.0 model, including formal and informal resources arranged along an integrated continuum that is comprised of nine steps. These steps range from low intensity (e.g., self-directed educational materials) to high intensity (e.g., crisis care, comprehensive case management). SC2.0 prioritizes client’s needs, preferences, readiness, informed choice, and strengths.. This approach recognizes that one service type is not suitable for everyone, and that people engage best with resources that meet their needs and preferences when they are ready. SC2.0 is comprised of nine core components, five of which are related to design and improvement of the model and four that are related to the client’s care experience. A set of practice recommendations (akin to an implementation guide) was developed by Stepped Care Solutions, developers of the SC2.0 model, and the Mental Health Commission of Canada (Stepped Care Solutions, Mental Health Commission of Canada: Stepped Care 2.0 Practice Recommendations, unpublished). Within the implementation guide, each core component is detailed, with a set of minimum and optimal practice recommendations for implementers to consider.

One of the core components of an SC2.0 model is the integration of a One-at-a-Time (OAAT) approach in the delivery of services. An OAAT therapeutic approach, more commonly referred to as single-session therapy, centers the client’s top-of-mind concern, and treats each client encounter as a whole and an opportunity to promote hope, growth, and change [2]. OAAT and single-session therapy are a form of brief therapy, and often follows a structured approach for the session [3]. Specifically, at the beginning of each encounter, the provider explores the client’s goal for the session and centers the discussion on what they hope to achieve. Through a guided conversation, the client and provider collaboratively consider services, supports, and actions in moving clients closer to their goal. Each OAAT therapeutic conversation draws on the strengths and capacity of the client, as well as resources available to them. Clients leave each session with an individualized wellness plan, outlining agreed-upon next steps to best address their top-of-mind concern [3, 4]. While beneficial to integrate into multiple levels of care (e.g., using an OAAT approach in peer support), an OAAT approach is most often seen in the individualized counselling step of the model through the use of single-session therapy methodology. The flexibility of an OAAT approach allows for the delivery of services virtually or in-person by a trained (e.g., [4]) mental healthcare professional. Importantly, while OAAT therapy is delivered as a standalone session, clients can return for additional sessions as needed. Removing the need to book multiple ongoing sessions for every client, and pivoting from lengthy assessments to a care-first approach, an OAAT therapy framework has the potential to reduce wait lists for 1:1 support [5], and increase access to care [1].

Like most other jurisdictions in Canada [6], the province of New Brunswick (NB), situated on the east coast of Canada with a population of over 800,000 individuals [7], was experiencing lengthy wait-times within addiction and mental health services. NB’s addiction and mental health services are provided through a partnership between the Department of Health (DoH) and two Regional Health Authorities: Vitalité Health Network and Horizon Health Network. The DoH is responsible for planning, funding, and monitoring services, while Horizon and Vitalité are responsible for operationalizing and delivering addiction and mental health services. Numerous non-governmental organizations and Indigenous communities also play a key role in service delivery.

With difficulty accessing services and challenges navigating the system being raised as the most frequent criticisms, the DoH conducted formal and informal reviews of community addiction and mental health services in consultation with providers, administrators, service users, families, and community partners between 2014 and 2020 [8]. As outlined in the review, community services are offered across the province through 14 Community Addiction and Mental Health Centres (CAMHCs) and numerous satellite offices. Services include screening for addiction and mental health-related issues, assessment, individual counselling, case management, psychoeducational and therapeutic group programs, and intensive services (e.g., mobile crisis intervention and Flexible Assertive Community Treatment). The province also has inpatient services available through psychiatric units that operate within general hospitals and specialized tertiary psychiatric hospitals, addiction centres, short-term treatment programs, and longer-term live-in transitional services [9].

Information and recommendations from the review of services performed by the DoH, as well as other priorities identified by the Government of NB, formed part of the Five-Year Interdepartmental Addiction and Mental Health Action Plan, which was launched in February of 2021 [8]. This plan aims to address the increasing demand for substance use and mental health services by focusing on five key areas: 1) improving population health; 2) improving access to services; 3) reducing drug-related harms; 4) intervening earlier; and 5) matching individuals to an appropriate level of care. Implementing a SC2.0 continuum of services and providing rapid access to services through the delivery of OAAT therapy were cornerstones of the Action Plan that aligned with key areas 2 and 5.

Appreciating the importance of successful healthcare implementations, and aligning with implementation science values, evaluation of the process and outcomes associated with implementing OAAT therapy within the context of SC2.0 was made a priority by the Government of NB and DoH. Implementation science research over the last 20 years has resulted in the development and wide-spread use of evidence-based frameworks, theories, and strategies to help promote successful and sustainable implementations. For instance, the National Implementation Research Network (NIRN) established a series of implementation stages and associated activities based on a review of approximately 800 implementation science studies [10]. These stages and corresponding processes have been applied in various implementations in the public sector, and in settings such as the education system [11], child welfare system [12], and healthcare system [13].

We had three objectives for the current study: 1) explicate the process of implementing OAAT therapy in CAMHCs (adult settings) as it aligned to select evidence-based implementation frameworks and strategies; 2) explore provider perceptions of organizational readiness during the implementation process; and 3) evaluate initial system outcomes and client experience with service delivery associated with the implementation. Through the retrospective application of select frameworks and strategies, we aimed to support other implementers in understanding important processes associated with improving access to addiction and mental healthcare that are aligned with established implementation frameworks, and provide conceptualization of how evidence-based frameworks and strategies can be applied to large-scale change and the implementation of OAAT therapy.

## Methods

### Procedures

#### Objective 1: explicating alignment with implementation frameworks and strategies

The DoH planned, prepared, and executed the rollout of OAAT therapy in a manner that aligned with evidence-based implementation frameworks, theories, and strategies, including: 1) Stages of Implementation (SI), one of five frameworks included in the Active Implementation Frameworks (AIF-SI), that defines four implementation stages (exploration, installation, initial implementation, and full implementation) [10]; 2) Consolidated Framework for Implementation Research (CFIR) [14], which is an implementation determinant framework that outlines five domains (intervention characteristics, outer setting, inner setting, individual characteristics, and process of implementation) and several constructs (e.g., adaptability, patient needs and resources, implementation climate) that influence implementation; and 3) Expert Recommendations for Implementing Change (ERIC), which identifies 73 implementation strategies derived from expert consensus [15]. The DoH regularly consulted with implementation scientists from our team and utilized implementation tools recommended by our experts. These implementation tools were developed and tailored to implementing OAAT therapy within the context of SC2.0. While enacted prospectively in some instances, we mapped the application of implementation frameworks and strategies retrospectively given that the prospective implementation plan was not developed and enacted in an intentional and rigorous manner.

**Fig. 1:**
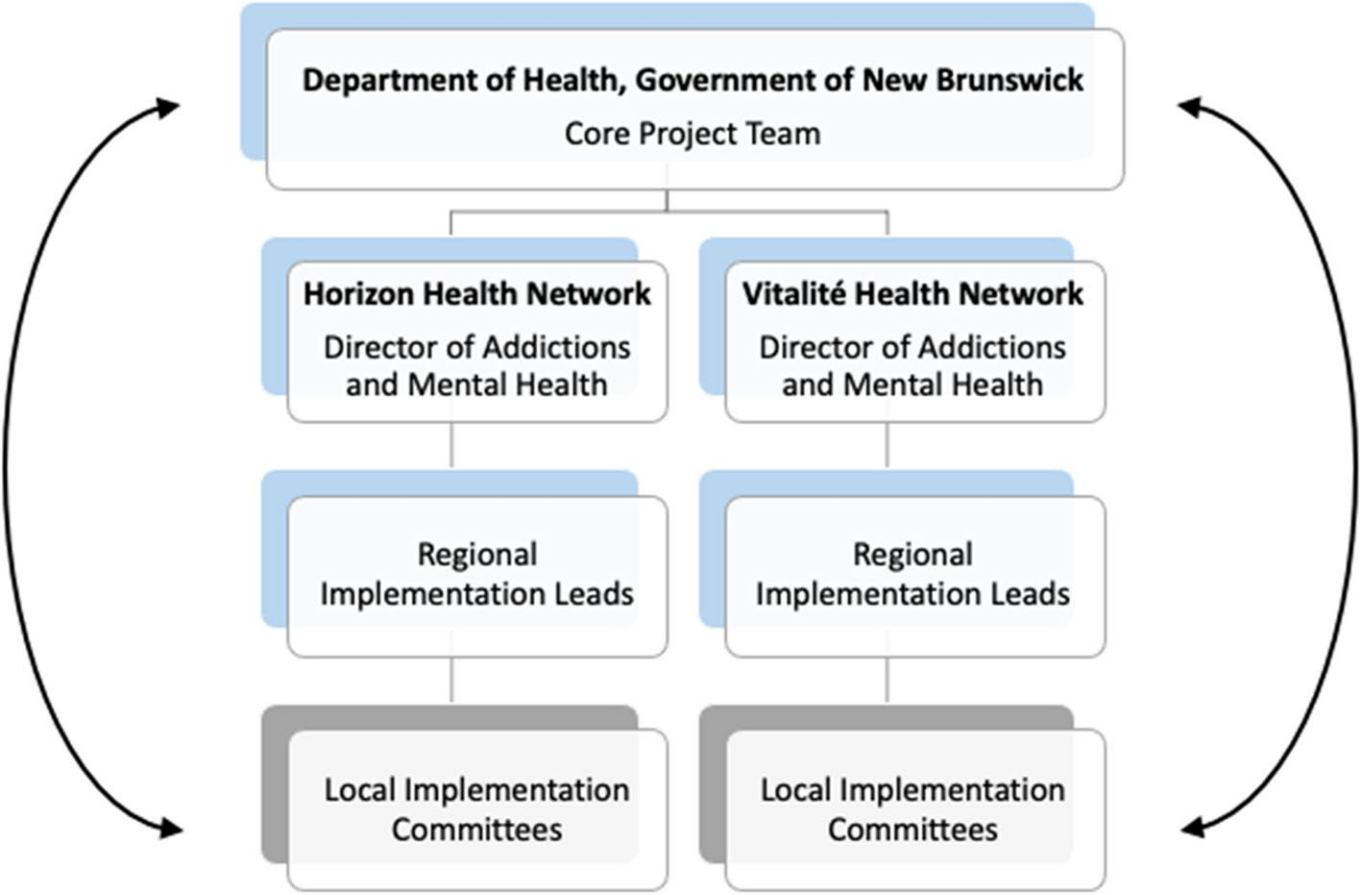
Flow of information from core project team to providers implementing OAAT therapy in their practice. *Note*: Blue squares denote members of the provincial working group

The process of implementing OAAT therapy in CAMHCs was documented by the provincial working group, equivalent to an implementation team (refer to Fig. 1). The provincial working group was comprised of the core project team (the project lead, subject-matter expert, project manager, and change management specialist), regional implementation leads (regional managers), and the directors of Addiction and Mental Health Services for Horizon and Vitalité Health Networks. Further, regional implementation leads formed local implementation committees with providers from the CAMHCs.

Our research team interviewed the core project team (Health Research Ethics Board Approval Ref #2022.048) to better understand the process of implementing OAAT therapy in the adult CAMHCs. These semi-structured interviews included questions on logistical implementation planning, preparation, and execution, successes and challenges encountered, and upcoming measures for sustainability.

In addition to interview data, other data sources included planning documents, progress reports and field notes from the provincial working group, government reports and presentations, and documented themes of employee questions and comments at various implementation milestones. Data from the DoH outlining details and outcomes of the implementation were shared with the partnering institutions, including Horizon and Vitalité Health Networks, Stepped Care Solutions, and Memorial University of Newfoundland.

#### Objective 2: exploring providers’ readiness and attitudes

Providers serving adult clients across NB were recruited from Vitalité and Horizon Health Networks. Eligible participants included mental health professionals from diverse backgrounds (e.g., social workers, nurses, psychologists). Participants were recruited via email and knowledge transfer meetings used to inform staff about asynchronous online courses in OAAT therapy within the context of SC2.0 and the SC2.0 model, and the associated research project. The recruitment email was circulated by managers and directors of the CAMHCs and contained a link to an informed consent form hosted on the survey platform Qualtrics [16]. Participants who did not consent to participate in research were redirected to receive immediate access to the asynchronous courses. Providers who consented to participate in the study completed a pre-course survey (T1) that included demographics. Participants then completed the online OAAT therapy course followed by the online SC2.0 course in a fixed order. The duration of each training was approximately 3 to 5 h. Additional surveys (T2) were distributed once providers completed the online courses. These surveys measured organizational readiness, and acceptability, appropriateness and feasibility of implementing SC2.0 in NB. Finally, participants received a follow-up survey (T3) one-month after completing the courses that contained a measure of organizational readiness (refer to Table 1 for relevant surveys and measures). OAAT therapy is one core component of the SC2.0 model, and the first core component that the DoH in NB chose to implement on their path to adopting a provincial SC2.0 model. As such, measures of acceptability, appropriateness, feasibility, and readiness for SC2.0 were used as a proxy for understanding acceptability, appropriateness, feasibility, and readiness of implementing OAAT within the context of SC2.0.

**Table 1:** Schedule of study assessments

Measures and Subscales	Pre-course (T1)	Post-course (T2)	One-month follow-up (T3)
Demographics	**X**		
Acceptability, Appropriateness, and Feasibility of Intervention Measure (AIM/IAM/FIM)		**X**	
Readiness for Organizational Change (ROC)		**X**	
Readiness Diagnostic Scale (RDS)			**X**

While other measures were completed at each time-point, only relevant measures are listed and discussed in this manuscript

Each survey took approximately 30 min to complete, and participation was incentivized with three $20 gift cards (e.g., Amazon). All materials were made available in English and French. This study was approved by Horizon and Vitalité Health Networks ethics boards (Ref# 2021–3015 and Ref# 2957, respectively) and the Newfoundland and Labrador Health Research Ethics Board (Ref# 2021.094).

#### Objective 3: evaluating initial system outcomes and client experience with service delivery

*System Performance Indicators:* Key performance indicators were abstracted through the provincial Client Service Delivery System (CSDS). Providers completed documentation in the CSDS system after each OAAT therapy session with a client to track the number of clients using this service. System indicators included the number of OAAT therapy sessions delivered between October 2021 and March 2022; waitlisted clients six months before the implementation and during the implementation period; and clients who presented for more than one session. Key performance indicators were frequently reviewed to monitor client access to services.

*Client Satisfaction Survey:* Adults who availed of an OAAT therapy session in one of the 14 CAMHCs across NB were eligible to participate. Administrative or clinical staff offered clients the opportunity to complete a satisfaction survey after receiving an OAAT therapy session. Participation was voluntary, and consenting clients received a paper survey that took approximately 5 min to complete. Clients returned the survey to the provider or administrative staff. Surveys were administered for a 6-month period after the implementation of OAAT therapy (October 2021 to March 2022).

### Measures

#### Objective 1: explicating alignment with implementation frameworks and strategies

Data sources included: 1) semi-structured interviews with the core project team; 2) government reports and presentations; 3) field notes, meeting minutes, and planning documents; 4) documented discussions on successes, challenges, and lessons learned; and 5) documentation and notes on recurring common questions and comments throughout the implementation.

#### Objective 2: exploring providers’ readiness and attitudes

Providers’ perceptions of the acceptability, appropriateness, and feasibility of implementing SC2.0 were determined using the 12-item *Acceptability of Intervention, Intervention Appropriateness, and Feasibility of Intervention Measures* (AIM/IAM/FIM) [17]. Participants rated their level of agreement with each statement on a 5-point Likert scale with anchors at “1 = Strongly disagree” and “5 = Strongly agree.” Mean values were calculated for each subscale with higher means suggesting greater acceptability, appropriateness, or feasibility. Cronbach’s alphas of the AIM/IAM/FIM in the current study were 0.95, 0.93, and 0.92, respectively.

We measured providers’ perceptions of organizational readiness for change using the *Readiness for Organizational Change* (ROC) Scale [18]. Participants responded to 25-items using a 7-point Likert scale with anchors at “1 = Strongly disagree,” and “7 = Strongly agree.” The measure contains four subscales, including: 1) appropriateness of SC2.0; 2) management support for implementing SC2.0; 3) change efficacy; and 4) personal benefit from SC2.0. The arithmetic mean was calculated for the total scale and each subscale, with higher values suggesting greater readiness. Cronbach’s alpha in the current sample was 0.91, 0.89, 0.82, and 0.88 for appropriateness, management support, change efficacy, and personal benefits, respectively.

Organizational readiness to implement SC2.0 was assessed using the *Readiness Diagnostic Scale* (RDS), which was adapted from the Readiness for Integrated Care Questionnaire [19] by the developer for use in this research initiative. Adaptations included reducing the measure from 82 to 51 items, and changing language around “integrated care” to “Stepped Care 2.0”. RDS items were rated on a 7-point Likert scale with anchors of “1 = Strongly disagree,” and “7 = Strongly agree.”. The RDS also contains 18 subscales, which are detailed and described in Additional file 1:  Appendix A. The arithmetic mean was calculated for the total scale and each subscale, with higher values suggesting greater readiness. Within the current sample, Cronbach’s alpha for the RDS was 0.98.

#### Objective 3: evaluating initial system outcomes and client experience with service delivery

*Key performance indicators* included the number of: OAAT therapy sessions delivered, discrete client visits, and waitlisted clients. Of note, the DoH upgraded the system for tracking waitlists shortly before the implementation of OAAT therapy. As a result, waitlist data between April 2021 to July 2021 may not be as accurate as the subsequent data.

*Client satisfaction* was measured through a survey developed by the CAMHCs. The survey was adapted from a client satisfaction survey used in the Newfoundland and Labrador demonstration project with the Mental Health Commission of Canada [20], and consultation with field experts in OAAT therapy and SC2.0. The survey consisted of five items that were rated on a 5-point Likert scale (refer to Additional file 1: Appendix B for questionnaire). No demographic information was collected in the survey to preserve confidentiality.

### Data analysis plan

#### Objective 1: explicating alignment with implementation frameworks and strategies

Semi-structured interviews were thematically analyzed by the research team (LHL, JAR). Emerging themes, detailing the implementation process, successes, challenges, and outcomes, were iteratively refined with the core project team (KB, MM, BG) until consensus was reached. Findings were also triangulated with government reports and presentations, notes and meeting minutes, and documentation of successes, challenges, and lessons learned.

Key implementation activities and processes discussed in interviews with the core project team were detailed in chronological order. The research team reviewed the order of events in comparison to the AIF-SI, CFIR domains, and ERIC strategies. Based on this review, the research team retrospectively determined which activities and processes aligned with the frameworks and strategies. Alignment was determined based on the definitions or criteria of the stages, domains and constructs, and strategies included in the AIF-SI [10], CFIR [14], and ERIC [15], respectively.

#### Objective 2: exploring providers’ readiness and attitudes

Missing data analyses were conducted, and data were determined to be missing at random. Imputation for missing data was not conducted given that the analytical approach for this objective was primarily descriptive in nature. Descriptive statistics and frequencies were performed to describe readiness, acceptability, appropriateness, and feasibility of implementing the SC2.0 model. Analyses were conducted using IBM SPSS Version 25 [21].

#### Objective 3: evaluating initial system outcomes and client experience with service delivery

Descriptive statistics were used to describe system performance indicators, including number of OAAT therapy sessions delivered, waitlist reductions, and number of presenting clients, as well as clients’ satisfaction with OAAT therapy. Additionally, comments in the satisfaction surveys were coded by one author using thematic coding analysis (SF) [22]. Comments were excluded from the analysis if they did not pertain to the therapy session. The remaining quotes were organized using a broad schema of positive, negative and neutral comments, and were assigned a code based on the messages provided by the client (e.g., “I feel much lighter now then I did when I walked in. Thank you!” were divided into two codes: 1) “Improvement in wellbeing/OAAT therapy session was helpful” and 2) “client appreciation for OAAT therapy session”). Resulting themes were reviewed and discussed with the team for consistency and conciseness.

## Results

### Objective 1: explicating alignment with implementation frameworks and strategies

Additional file 1: Appendix C depicts a detailed outline of the steps and activities that were enacted in NB, which align with implementation frameworks and strategies. The following sections explore key implementation activities that correspond to the AIF-SI. The majority of work that occurred within the timeframe of this research project aligned with the installation and initial implementation stages.

#### Exploration stage

The exploration stage began with a review of services, and consultations with providers and clients between 2014 and 2020, which led to the development of the 5-Year Action Plan. The DoH explored models to facilitate rapid access to services and provide an integrated continuum of care, and determined that the implementation of a provincial SC2.0 model, beginning with the delivery of OAAT therapy, would best meet the needs of clients, providers, and the system. NB’s Action Plan was released in 2021 [8].

Campbellton, a small city in NB, started piloting OAAT therapy in their CAMHC in November 2020. Establishing an early adopter site and piloting the service assisted the DoH in determining successful practices, trialing procedures and documents (e.g., a client satisfaction survey), and helped identify potential areas of concern for a large-scale province-wide implementation.

The DoH and Horizon and Vitalité Health Networks forged strong partnerships with a not-for-profit organization, Stepped Care Solutions, and an academic institution, Memorial University of Newfoundland in January 2021 as part of a larger evaluation of the implementation of SC2.0 across several Atlantic-Canadian provinces. These partnerships allowed for the evaluation of provider attitudes and readiness for change, and ensured that the DoH received data on implementation indicators to iteratively improve the implementation process.

The DoH formed the core project team in January 2021, and hired a provincial change management specialist in June 2021, who had expertise in change management methodologies [23]. The change management specialist was hired to enhance organizational readiness, address potential resistance in the implementation process, and prepare stakeholders at all levels for sustainable change. The core project team included membership from the project team lead (Director of Addiction and Mental Health Adult Services with the Government of NB), subject-matter expert (a clinical lead within the DoH), project manager, and change management specialist. With a mandate to lead the implementation of OAAT therapy in all Health Zones across the province, the core project team was reported to be a critical factor to oversee the successful implementation, including adoption, reinforcement and sustainability of the new service.

The DoH established a provincial working group prior to initiation of the installation stage that effectively operated as an implementation team. The local implementation committees promoted the review of successes and concerns, and determined implementation logistics at the local level. As outlined in Fig. 1, the structure of teams fostered a feedback loop between the provincial working group and the local implementation teams. Clinical leads were also hired to coordinate services in their health zone, provide clinical support to providers delivering OAAT therapy, and promote the role of OAAT therapy in a SC2.0 continuum of services.

#### Installation stage

Guided by the core project team, the provincial working group met weekly to: 1) develop, review, and continuously adapt the formal project plan, which included change management, training, and communication strategies; 2) review system-related processes and operational changes required to effectively implement OAAT therapy (i.e., intake processes, information management changes, and provider documentation); and 3) assess organizational readiness and mitigation strategies for anticipated barriers. The established implementation and change management strategy included six components: 1) define roles and responsibilities, and develop a shared vision and understanding of project context, objectives, and anticipated benefits and barriers; 2) analyze potential barriers and readiness on an ongoing basis; 3) prioritize engagement with managers and implementation leads to ensure effective leadership; 4) train impacted stakeholders to deliver OAAT therapy with clients; 5) continuously assess and address causes of resistance to adoption; and 6) integrate sustainability and reinforcement measures into all aspects of implementation planning and execution.

The training strategy commenced with education sessions for providers to ensure they had opportunities to learn about system changes, and voice their questions and concerns. Next, over 375 staff completed online asynchronous courses in SC2.0 and OAAT therapy, with 170 (45%) participating in research. These courses allowed staff to better understand the upcoming changes in the system and how their role would be affected by the implementation. After completing the asynchronous courses, approximately 300 adult providers attended a 2-day face-to-face training with field experts in OAAT therapy to further their knowledge and abilities, and receive coaching. Finally, a communication plan was developed that outlined documents to disseminate to stakeholders. Execution of training and communication plans involved significant direction and participation from leadership, particularly the project lead.

The change management specialist documented and categorized successes and concerns that surfaced from the feedback loop to better understand readiness, circumvent potential barriers, and strengthen likely facilitators. For instance, staff resistance towards completing training and adopting an OAAT therapeutic approach in CAMHCs was noted, which led to the provincial working group communicating the importance of having managers and clinical champions lead by example. This led managers and champions to complete the OAAT therapy training first, which was viewed as a facilitator of training uptake.

The provincial working group developed new administrative service guidelines in preparation to implement OAAT therapy that were distributed to providers for review and reference. The guidelines included: 1) restructuring sessions to follow an OAAT therapeutic approach; 2) streamlining clinical documentation; 3) updating referral processes, allowing for self-referrals without necessitating an intake process; and 4) information management system changes.

#### Initial implementation stage

The provincial working group set a timeline from October 2021 to March 2022 to achieve initial implementation. Providers began piloting OAAT therapy in their practice between August and October 2021, with all CAMHCs offering OAAT therapy services by October. Between October 2021 and March 2022, the project team worked to iteratively evaluate and improve the delivery of OAAT therapy services. During this time, each CAMHC team reviewed and consolidated their waitlist, as some waitlisted clients may have no longer needed services.

The project team collaborated with the provincial working group to distribute a survey to evaluate client satisfaction with the OAAT therapy session. The change management specialist continued to monitor readiness as services were implemented, and tracked successes and concerns. Moreover, program champions who were experiencing successes in the delivery of OAAT therapy services were provided opportunities to present to other clinics in order to facilitate the adoption of successful practices.

Interviews were conducted by the core project team with each of the 14 CAMHC teams at the conclusion of the initial implementation phase and analyzed thematically. Interviews between the core project team and CAMHC teams fostered continuous implementation improvement, and highlighted: 1) inconsistencies between the pre-existing operational guidelines and new administrative guidelines; 2) capacity challenges relating to staffing, space, and training; and 3) difficulties changing perceptions held by clients, organizational partners, and providers that long-term therapy is a gold standard, even when therapeutic gains were no longer being achieved. Service guidelines underwent a second review to promote appropriate adaptation, improve the service delivery process, and enhance sustainability. The DoH worked with providers to foster continued change in culture, including developing language around the delivery of OAAT therapy that highlighted availability of additional sessions as merited.

#### Full implementation stage

The provincial working group shifted focus to sustainability as the initial implementation stage concluded. Key activities to reinforce sustainability included: 1) developing processes to train newly hired staff; 2) engaging in regular consultations between providers and clinical leads to address novel concerns, and provide sustained coaching; and 3) introducing a community of practice in Spring 2022. The community of practice fostered continued professional development, allowed for consultation with subject-matter experts, and provided a venue to highlight successes and address concerns.

The DoH is currently in the process of measuring fidelity in the delivery of OAAT therapy. This process will gather information from CAMHCs, and includes questionnaires, interviews with clinical leads and providers, and random chart audits. The goal is to ensure that providers adhere to principles of OAAT therapy when delivering care. Moreover, supervisors recently completed a supervisor-specific training to strengthen supervisory skills and develop more consistency in supporting providers delivering OAAT therapy.

### Objective 2: exploring providers’ readiness and attitudes

#### Sample characteristics

Adult providers (*N* = 170) enrolled in the study and completed surveys at time 1 and 2, and 109 providers completed the time 3 survey. Sample characteristics are summarized in Table 2. English was the preferred language of most participants (64.7%). Participants were employed within Horizon (56.5%) and Vitalité Health Networks (42.9%), with 0.6% endorsing another work setting (e.g., DoH), and on average, had worked in their current role for approximately 8 years.

**Table 2:** Summary of participant characteristics

Characteristic	Sample size (*N*)	Valid percent (%)
Language		
English	110	64.7
French	60	35.3
Organization of Work		
Horizon Health Network	96	56.5
Vitalité Health Network	73	42.9
Other	1	0.6
Work Setting		
Community Mental Health/Addiction Clinic	146	85.9
Hospital/Psychiatry Unit	13	7.6
Primary Care Clinic	7	4.1
Other	4	2.4
Community Setting		
Urban	103	60.6
Rural	67	39.4
Profession		
Social Work	85	50.0
Nursing	52	30.6
Business Admin/Admin	2	1.2
Psychology	7	4.1
Occupational Therapy	7	4.1
Counselling	4	2.4
Other	13	7.6
Level of Education		
Doctorate	2	1.2
Master’s	31	18.2
Baccalaureate	110	64.7
Professional Certificate/Diploma	21	12.4
Other	6	3.5
Role		
Provider	152	89.4
Clinic Coordinator/Clinical Lead	4	2.4
Manager	5	2.9
Manager/Provider	9	5.3

#### Acceptability, appropriateness, feasibility, and readiness outcomes

As highlighted in Table 3, providers agreed that the implementation of SC2.0, including OAAT therapy, was acceptable (*M* = 4.36, *SD* = 0.62), appropriate (*M* = 4.25, *SD* = 0.67), and feasible (*M* = 4.10, *SD* = 0.69). Additionally, participants felt that SC2.0 was appropriate for their organization (*M* = 5.83; *SD* = 0.94); management and senior leaders supported the implementation (*M* = 5.54*; SD* = 0.98)*;* SC2.0 could be successfully adapted for implementation within their practice (*M* = 5.32*; SD* = 1.41); and there will be personal benefits for implementing SC2.0 into practice (*M* = 5.80*, SD* = 1.22)*.* Overall, providers agreed that their organization was ready to implement a provincial stepped care model (*M* = 5.64*, SD* = 0.84).

**Table 3:** Descriptive statistics for measures of acceptability, appropriateness, feasibility, and readiness

Measures and Subscales	Mean (*M*)	Standard Deviation (*SD*)
Acceptability of Intervention Measure (AIM)	4.36	0.62
Intervention Appropriateness Measure (IAM)	4.25	0.67
Feasibility of Intervention Measure (FIM)	4.10	0.69
Readiness for Organizational Change (ROC)		
Appropriateness of SC2.0	5.83	0.94
Personal benefit from SC2.0	5.80	1.22
Management support for implementing SC2.0	5.54	0.98
Change Efficacy	5.32	1.41
Total ROC score	5.64	0.84
Readiness Diagnostic Scale (RDS)		
Compatibility	6.00	0.83
Observability	5.77	1.01
Program Champion	5.79	1.34
Priority	5.60	1.41
Simplicity	5.57	1.09
Culture	5.45	1.24
Leadership	5.42	1.46
Innovation Specific Knowledge and Skills	5.33	1.41
Relative Advantage	5.30	1.44
Supportive Climate	5.31	1.46
Ability to Pilot	5.21	1.53
Climate	5.25	1.28
Innovativeness	5.24	1.44
Intra-Organizational Relationships	5.01	1.69
Structure	5.06	1.44
Inter-Organizational Relationships	4.80	1.71
Staff Capacity	4.55	1.70
Resource Utilization	4.52	1.74

Evaluation of readiness through the RDS highlighted that providers agreed that SC2.0 is compatible with their organization and clients (*M* = 6.00*, SD* = 0.83), short-term results would be observable (*M* = 5.77*, SD* = 1.01), and program champions were present (*M* = 5.79*, SD* = 1.34). Conversely, providers somewhat agreed that their organization had sufficient staff capacity (*M* = 4.55*, SD* = 1.70) and communication and relationships with other organizations to effectively implement OAAT therapy within the context of SC2.0 (*M* = 4.80, *SD* = 1.71), refer to Table 3.

### Objective 3: evaluating initial system outcomes and client experience with service delivery

#### Key system performance indicators

**Fig. 2:**
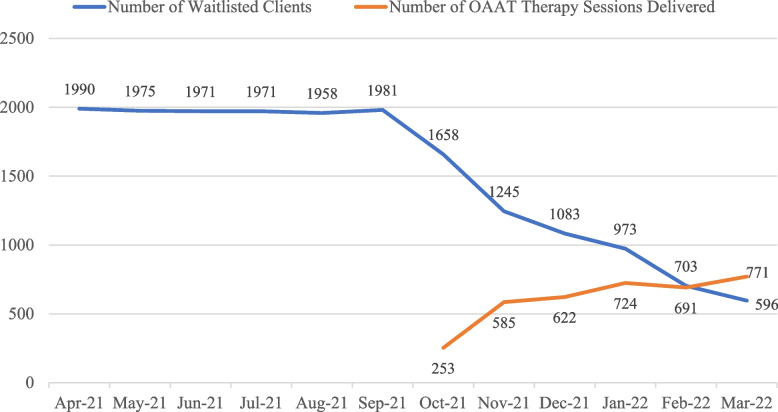
Number of waitlisted clients and OAAT therapy sessions delievered by month. *Note*: implementation period of OAAT therapy occurred between October 2021 and March 2022

As detailed in Fig. 2, a total of 3,646 OAAT therapy sessions were offered between October 2021 and March 2022 across the 14 CAMHCs. Among the 3,213 clients who presented for an OAAT therapy session, 89.5% of clients utilized only one session, with 8.3% returning for a second session, and the remaining 2.2% returning for three to five additional sessions. The most common presenting concerns were related to symptoms of depressed and anxious mood. Nearly 2,000 clients were waitlisted to receive individualized therapy between April and September 2021 (pre-implementation). Through the delivery of OAAT therapy sessions, review and consolidation of the waitlist, and upgrades to the waitlist tracking system, the waitlist was reduced by 64.1% from October 2021 to March 2022 (refer to Fig. 2). Since the completion of the initial implementation stage, an additional 4,718 sessions were delivered by October 2022.

#### Client satisfaction surveys

A total of 1,240 clients (34.0% response rate) completed the satisfaction survey after receiving an OAAT therapy session. Just over 90% of clients (*n* = 1147) reported feeling satisfied or very satisfied that the session helped them develop a plan to address their immediate concern. Further, 75% of clients indicated that they were worried or very worried about their concern before attending an OAAT therapy session which was reduced to 16% endorsing this level of concern following session completion. In total, 58% of clients indicated feeling confident or very confident to deal with their concern after the session. Finally, 92% of clients reported feeling satisfied or very satisfied that their concern was addressed during the session.

Open-ended comments were provided by 224 clients and resulted in the synthesis of six themes, refer to Table 4. Themes included: 1) The OAAT therapy session was a positive experience (*n* = 112); 2) Clients appreciated the OAAT therapy session (*n* = 52); 3) Clients reported improved wellbeing and/or that the session was helpful (*n* = 43); 4) OAAT therapy sessions provided clients with an actionable plan, knowledge, tools and/or resources (*n* = 32); 5) Clients valued quick, open, and flexible access to OAAT therapy (*n* = 17); and 6) The OAAT therapy session did not meet client needs among a small portion of clients (*n* = 15).

**Table 4:** Results of thematic analysis derived from client satisfaction survey comments

**Description of review finding**	**Supporting quotation**	**Number of endorsements** (*N*** = 224)**
**Theme 1: An OAAT therapy session provided clients with a good plan, knowledge, tools and/or resources**		
Clients felt the session provided them with a good plan, knowledge, tools and/or resources	*Feel like I have a good plan—tips provided/suggestions—resources provided*	*(n* = *32)*
**Theme 2: The OAAT therapy session was a positive experience for clients**		
Clients described their OAAT therapy session as a positive experience (i.e., a pleasant experience with a specific provider, feeling satisfied with the session, being able to voice concerns and feeling validated.)	*I am very happy I came. It was well worth it.:)*	*(n* = *112)*
	*The therapist was very helpful, empathetic, and knowledgeable*	
	*Great energy. Great communicator, enjoyed the time with her*	
**Theme 3: Client appreciation for the OAAT therapy session**		
Clients expressed appreciation for the OAAT therapy session	*Thank you for offering this service, it is really appreciated*	*(n* = *52)*
**Theme 4: Client’s valued quick, open, and flexible access to OAAT therapy**		
Clients valued OAAT therapy and the quick, open, and flexible access to care that it offers, such as multiple sessions, online or telephone appointments, and the lack of wait time	*I love this new form of "therapy/consultation"!*	*(n* = *17)*
	*Super excited about new service! Was able to see someone fast!*	
	*Love the fact that if I still need a session I can call back for an appointment*	
**Theme 5: Clients report improved wellbeing and/ or that the OAAT therapy session was helpful**		
Clients reported an improvement in their wellbeing immediately after the OAAT session and/ or describe the session as helpful	*I feel much better after this meeting.:)*	*(n* = *43)*
	*This session has helped me*	
	*I feel much lighter now than I did when I walked i*	
**Theme 6: OAAT therapy session did not meet client’s needs**		
Clients reported that the OAAT therapy session did not meet their needs, such as they were disappointed, the provider was not a good fit, or that they require regular and set appointments with the same provider	*This was only one session. These people are not miracle workers. Not going to be able to fix someone in 30 min but it was nice to talk to someone. I just feel I needed more time to talk*	*(n* = *15)*
	*I need the same person to listen not multiple*	
	*I'm at a loss. Completely*	

## Discussion

Individuals living in NB were experiencing protracted wait times and difficulties accessing the addiction and mental healthcare system at the time the 5-Year Action Plan was released. It was critical for NB to implement OAAT therapy within the context of a provincial stepped care model given the increased demand for services [8], and the notion that long wait-times can lead to deterioration of mental health [24]. Our study documented the process of implementing OAAT therapy in NB, along with concomitant changes in key performance indicators and client experience with service delivery.

Aligning with established frameworks and strategies, the DoH prioritized engagement of provincial, regional, and organizational leaders in the provincial working group and in the delivery of communication, education sessions, and other employee- and public-facing engagement activities. It is possible that the stable presence of engaged leaders helped to foster success in implementation determinants [25], given that leadership engagement is associated with an increase in the likelihood of implementation success [26], as well as improved organizational culture [27], staff engagement [28] and learning climate [14]. For instance, engagement of middle-managers (i.e., those supervising front-line staff) in the provincial working group helped to promote a collaborative climate for change and facilitate an effective feedback loop of communication between upper management and front-line staff. Moreover, the effective feedback loop ensured that incongruence between an OAAT therapeutic approach and organizational policies and procedures could be communicated from front-line staff, prompting further review of the organizational guidelines. Leadership representation from the two health authorities and DoH also helped facilitate policy change (i.e., around referrals).

Preliminary results suggested that providers felt their organization was ready to implement OAAT therapy within the context of the SC2.0 framework, and that the approach was appropriate, acceptable, and feasible. Aligning with previous implementation work [29], it is plausible that the DoH’s careful review of existing services, and consideration of program and implementation site indicators (i.e., evidence, fit, usability, and capacity) enhanced feasibility and acceptability within this implementation.

Areas of growth were noted among the positive outcomes of the readiness assessments. For instance, providers indicated that staffing capacity may benefit from continued monitoring, as the average score fell within slight agreement. It is not surprising that we observed staffing capacity as one of the lowest scores of readiness for this group, given the increased strain and burden that healthcare staff have experienced during the COVID-19 pandemic [30, 31], in an already overwhelmed addiction and mental healthcare system [32, 33]. It will be imperative to continue to ensure sufficient staffing and resourcing as sustainability becomes the primary focus.

The implementation of OAAT therapy was accompanied by a reduction in people awaiting care and high levels of client satisfaction. With previous literature noting declines in client wellbeing while waiting to access addiction and mental health services [24], it was encouraging that clients were able to attend an appointment when they needed care. Our findings are also consistent with outcomes from other clinics that have implemented single-session or drop-in services [5, 34, 35], as waitlists and wait times were reduced, facilitating quicker access to services. Moreover, client feedback from our study aligns with client feedback from previous research, as clients were satisfied with the session they received, felt that it was beneficial for their current problem, and felt that the model of delivery was a good fit [5, 36]. While most clients were satisfied with the session they received, some indicated that one session was not enough. This finding is consistent with other client experiences [37] and work by field experts noting that OAAT therapy is not a suitable approach for everyone [38], further highlighting the importance of the SC2.0 integrated continuum of care.

### Limitations

The results detailed in this manuscript must be interpreted in light of several limitations. First, an observational design was used, and causation cannot be inferred. Second, implementation strategies were aligned with theories and frameworks post-hoc rather than through the purposeful development of a logic model a-priori. As such, it is difficult to discern the true benefit that these frameworks could have in guiding such an implementation. It is important to note that several team-members brought implementation science expertise to the planning and initial-implementation phases (e.g., Memorial University of Newfoundland, Stepped Care Solutions). Third, despite its importance [39], fidelity to practicing with an OAAT therapeutic approach was not measured throughout the initial implementation stage, which may impact the validity of results observed. Fourth, a lived experience advisory committee was not assembled or consulted during the installation and initial implementation steps despite their importance to help bridge the gap between research and real-world utility while improving sustainability and equitability of healthcare services [40]. Fifth, findings on client satisfaction with OAAT therapy should be interpreted with caution due to the low response rate and potential risk of bias. Finally, provider perceptions and client experience with service delivery were measured using self-report and may be subject to response bias and should be interpreted with caution. For example, highly motivated providers and clients may have been more likely to complete surveys. Tempering this concern, our response rate among providers across the province was high (~ 50%) and likely representative. Similarly, client surveys were anonymous, short in length, and provided immediately following OAAT therapy sessions which likely increased response rate.

## Conclusions

To our knowledge, this is the first study to explicate the process of implementing a provincial practice change to facilitate rapid access to mental healthcare in alignment with contemporary implementation frameworks and strategies. With no known research on the pragmatic and logistical considerations to implementing an OAAT therapeutic approach to services, our study outlines important steps and considerations for provinces and organizations looking to enact large-scale change and facilitate rapid access to addiction and mental healthcare services. Although specific to the implementation of OAAT therapy services within NB CAMHCs, it is our hope that the implementation process outlined in this report can inform other jurisdictions looking to implement such services.

### Supplementary Information

**Additional file 1.**

## Data Availability

De-identified data will be made available to researchers who provide a methodologically sound proposal for the purpose of achieving the aims of the approved proposal. Data sharing will be enacted with a data-transfer agreement between the sending and receiving institutions. Proposals should be directed to the corresponding author.

## Abbreviations

AIM/IAM/FIM: Acceptability of Intervention, Intervention Appropriateness, and Feasibility of Intervention Measures
AIF-IS: Active Implementation Framework-Implementation Stages
CAMHCs: Community addiction and mental health clinics
CFIR: Consolidated Framework for Implementation Research
CSDS: Client Service Delivery System
DoH: Department of Health
ERIC: Expert Recommendation for Implementing Change
NB: New Brunswick
OAAT therapy: One-at-a-Time therapy
RDS: Readiness diagnostic survey
ROC: Readiness for organizational change
SC2.0: Stepped Care 2.0
