# Supplementary material for: Implementing One-at-a-Time Therapy in community addiction and mental health centres: a retrospective exploration of the implementation process and initial outcomes
Source: BMC Health Serv Res. 2023 Sep 12;23:982. doi: 10.1186/s12913-023-09923-5 (PMC10496188; doi:10.1186/s12913-023-09923-5)
Supplement: Supplementary file 1 — Additional file 1. [file 12913_2023_9923_MOESM1_ESM.docx]

Additional file 1: Appendix A

*RDS Subscales and Descriptions*

| **RDS Subscale** | **Subscale Description** |
| --- | --- |
| Ability to Pilot | The degree to which SC2.0 can be tested and improved in smaller settings before a full-scale implementation. |
| Climate | The overall feeling and tone within the organization. |
| Compatibility | The fit between SC2.0 and how the organization operates. |
| Culture | The alignment between SC2.0 and the organization’s norms and values. |
| Innovativeness | The organization’s openness and willingness to change. |
| Inter-Organizational Relationships | The organization’s relationships with other organizations and stakeholders implementing SC2.0. |
| Intra-Organizational Relationships | The relationships and level of coordination within the organization. |
| Knowledge and Skills | The skills and knowledge needed for staff to successfully use the SC2.0 model in their practice. |
| Leadership | The perceived effectiveness of the organization’s senior leaders. |
| Observability | The expectation to observe the intended impacts of SC2.0. |
| Priority | The importance of SC2.0 compared to the importance of other organizational operations. |
| Program Champion | The presence of individuals within the organization who are well connected and support the use of SC2.0 in their practice. |
| Relative Advantage | The advantage of SC2.0 compared to other innovative addiction and mental health models of care. |
| Resource Utilization | The organization’s ability to access and distribute financial resources to support SC2.0. |
| Simplicity | How simple or complex it will be to used SC2.0 in practice. |
| Staff Capacity | The workload of staff in relation to the capacity needed to carry out the SC2.0 model. |
| Structure | The structure and level of collaboration within the organization. |
| Supportive Climate | Presence of the necessary supports, processes, and resources to effectively enact SC2.0. |

Additional file 1: Appendix B

| 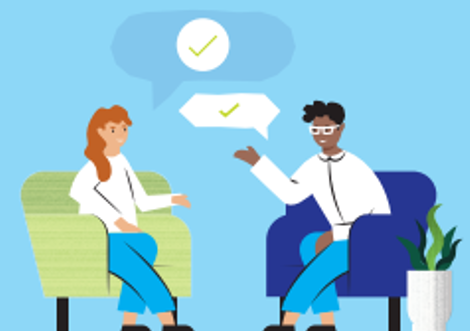 | **Adult Services**  **One-at-a-time Therapy Client Satisfaction Survey**  **Addiction and Mental Health Services** |
| --- | --- |
|  | *Small change leads to big change* |

1. **How upset / worried were you about your concerns /problems before this counseling session?**

| V*ery worried/upset* | *Worried/upset* | *Not sure* | *Somewhat worried/upset* | *Not worried/upset* |
| --- | --- | --- | --- | --- |
| 5 | 4 | 3 | 2 | 1 |

1. **How upset / worried are you about these concerns/problems after this counseling session?**

| V*ery worried/upset* | *Worried/upset* | *Not sure* | *Somewhat worried/upset* | *Not worried/upset* |
| --- | --- | --- | --- | --- |
| 5 | 4 | 3 | 2 | 1 |

1. **How confident do you presently feel in dealing with your concerns/problems?**

| *Very confident* | *Confident* | *Not sure* | *Somewhat confident* | *Not confident* |
| --- | --- | --- | --- | --- |
| 5 | 4 | 3 | 2 | 1 |

1. **How satisfied are you with how your concerns/problems were addressed?**

| *Very satisfied* | *Satisfied* | *Not sure* | *Somewhat satisfied* | *Not satisfied* |
| --- | --- | --- | --- | --- |
| 5 | 4 | 3 | 2 | 1 |

1. **How satisfied are you that this session helped you to develop a plan to address the concerns/problems?**

| *Very satisfied* | *Satisfied* | *Not sure* | *Somewhat satisfied* | *Not satisfied* |
| --- | --- | --- | --- | --- |
| 5 | 4 | 3 | 2 | 1 |

**Other Comments:**

|  |
| --- |
|  |
|  |

Additional file 1: Appendix C

| **Exploration Stage** | | | | | |
| --- | --- | --- | --- | --- | --- |
| **NB Implementation Step** | **Description of NB Implementation Step** | **Timeframe** | **NIRN Components** | **CFIR Domains and Constructs** | **ERIC Strategies** |
| Review of Provincial Addiction and Mental Health Services | Consultations were conducted with over 100 providers, clients, and family members, as part of a thorough review of outpatient services. Over 400 consultations were completed between the review of inpatient and outpatient services. Ultimately, the review of services led to a series of recommendations for an enhanced continuum of mental health and addiction care. | 2014 – 2020 | -Grow relationships with stakeholders with a variety of diverse perspectives. | Outer Setting:  -Patient Needs and Resources  Inner Setting:  -Implementation Climate (tension for change) | -Conduct local needs assessment  -Involve patients/consumers and family members |
| Review potential systems and models | The DoH considered various known systems, models, and frameworks for addiction and mental health services and explored the similarities and differences, as well as advantages and disadvantages. This review led to the selection of Stepped Care 2.0 as it has exceptional face validity and allows for a comprehensive and recovery-oriented continuum of services with varying levels of intensity. Further, the focuses on rapid access to care, promotion of evidence-based practices, and its applicability in the development of a large-scale province-wide service delivery framework were also key factors in its selection. | 2018 – 2020 | -Identify and learn about other potential programs to address change needed.  -Assess fit and feasibility of options to address change needed. | Inner Setting:  -Compatibility |  |
| Pilot OAAT therapy in Campbellton | OAAT therapy was piloted at a CAMHC in Campbellton. The pilot project assisted the DoH in determining successful practices, trialing procedures and documents (i.e., a client satisfaction survey), and helped identify potential areas of concern in a large-scale province-wide implementation. | Starting in November 2020 |  | Intervention Characteristics  -Trialability  -Relative advantage  -Adaptability | -Identify early adopters  -Stage implementation scale-up  -Model and simulate change |
| Formation of core project team | The core project team included membership from the project team lead (Director of Addiction and Mental Health Adult Services with the Government of NB), subject-matter expert (a clinical lead within the Department), project manager, and change management specialist. This team helped lead the implementation of OAAT therapy in all Health Zones across the province. The change management specialist was hired in June 2021 to enhance organizational readiness, address potential pain-points and resistance in the implementation process, and to help prepare stakeholders at all levels for sustainable change. | January 2021; June 2021 | -Begin to build implementation team | Inner Setting:  -Readiness for implementation  Process:  -Planning  -Engaging  -Executing  -Reflecting and evaluating | -Facilitation  -Recruit for leadership  -Use an implementation advisor |
| Partnerships with Stepped Care Solutions and Memorial University of Newfoundland | The Government of NB formed partnerships with Stepped Care Solutions and Memorial University of Newfoundland. These partnerships fostered implementation support and data collection on providers’ knowledge and beliefs about OAAT therapy and SC2.0, their self-efficacy, readiness/commitment, stage of change, work engagement and satisfaction, and competency in OAAT therapy and SC2.0. | January 2021 |  | Characteristics of Individuals  -Knowledge and beliefs about intervention  -Self-efficacy  -Stage of change  -Individual identification with the organization | -Develop academic partnerships |
| Develop formal implementation plan | The core implementation team developed the implementation plan, which guided the work of the provincial working group. The implementation plan included: the project charter and change management strategy, plan for training providers in OAAT therapy and Stepped Care 2.0, and communication plan. | January 2021 – August 2021 | Develop plan for implementation, develop communication processes and messages. | Process:  -Planning | -Develop a formal implementation blueprint  -Develop an implementation glossary  -Develop and implement tools for quality monitoring |
| Release of Provincial 5-Year Action Plan | The inter-departmental 5-year action plan for mental health and addiction care was released, and included priorities to create an integrated continuum of care and facilitate rapid access to services. | February 2021 | -Scan or assess what is currently in place to address the change needed.  -Identify the option to implement. | Inner Setting  -Readiness for Implementation (leadership engagement, available resources)  -Relative Priority | -Conduct local consensus discussions  -Obtain formal commitments  -Mandate change  -Access new funding |
| Assessing and tailoring data systems | Data collection by the Client Service Delivery System was assessed and revised to better capture data (e.g., number of OAAT therapy sessions delivered) on the impacts of implementation and to monitor delivery of services. | March 2021 – April 2021 | -Scan or assess what is currently in place to address the change needed  -Develop data systems (what data, how data will be collected, used, and shared). | Process:  -Planning | -Change record systems  -Develop and organize quality monitoring systems |
| Establish and educate Provincial Working Group (implementation team) | The provincial working group was comprised of various levels of leadership (directors, managers, implementation leads, and clinical leads) and the core implementation team (project lead, subject-matter expert, project management lead, and change management specialist).  The provincial working group was responsible for 1) developing, reviewing, and continuously adapting the formal project plan (including the change management, training, and communication strategies); 2) reviewing system processes and required changes to effectively implement OAAT therapy (i.e., intake processes, information management changes, and provider documentation); and 3) assessing organizational readiness and mitigation strategies for barriers. | May 2021 – June 2021 | -Built and revisit implementation team membership to ensure needed perspectives are present.  - Ensure implementation team has appropriate knowledge, skills, functions, and authority to support infrastructure development and improvement of the program/practice. | Inner Setting:  -Leadership Engagement  -Networks and Communications  Process:  -Engagement (formally appointed internal implementation leaders) | -Use advisory boards and workgroups  -Identify and prepare champions |
| **Installation Stage** | | | | | |
| **NB Implementation Step** | **Description of NB Implementation Step** | **Timeframe** | **NIRN Components** | **CFIR Domains and Constructs** | **ERIC Strategies** |
| Hold weekly Provincial Working Group meetings | Throughout the implementation stages, the provincial working group met weekly to fulfil their mandate with the formal project plan, system processes, and organizational readiness. | May 2021 – March 2022 | Convene implementation team regularly to use data to critically examine and improve implementation | Process:  -Reflecting and Evaluating | -Purposely re-examine the implementation  -Promote adaptability |
| Host information sessions with providers | Information sessions with the Director of Mental Health and Addiction for adult services and the change management specialist were delivered for providers to learn about system changes associated with implementing OAAT therapy in a provincial stepped care framework. These sessions also provided an opportunity for providers to voice their questions and concerns. | June 2021 – July 2021 | Provide initial training for practitioners | Inner Setting:  -Readiness for  Implementation (leadership engagement, available resources, access to knowledge and information) | Conduct educational meetings |
| Revise operational guidelines and processes | In preparation to implement OAAT therapy in CAMHCs, the provincial working group reviewed and revised organizational procedures and guidelines. Referral sources were changed so clients could self-refer to an OAAT therapy session without having to complete an intake-session. Documentation processes were also updated so providers could document the OAAT therapy session in the provincial Client Service Delivery System (CSDS). | June 2021 – October 2021 | -Review and refine needed policies and procedures.  - Develop data systems (what data, how data will be collected, used, and shared). | Inner setting:  -Compatibility | -Purposely re-examine the implementation  -Promote adaptability  -Develop and implement tools for quality monitoring  -Change record systems |
| Assess risk and readiness | Ongoing assessment of stakeholder readiness was completed by tracking questions and concerns about the implementation, and through check-ins with implementation leads. | June 2021 – March 2022 | Determine what is needed to optimize readiness and develop staff capacity, as well as organization and system changes needed. | Inner Setting:  -Readiness for  Implementation | -Assess for readiness and  Identify barriers and facilitators.  -Tailor strategies |
| Prepare implementation and hire clinical leads | Clinical leads helped coordinate OAAT therapy services in their Health Zone. This included providing clinical support and guidance to providers who deliver the service, building an understanding of the service within the local community, playing a leadership role in the OAAT therapy Community of Practice, and promoting the role of OAAT therapy in a SC2.0 continuum of services.  While Health Authorities began the hiring process of clinical leads in March 2021, these positions were filled during the installation and initial implementation stages, with all positions filled by August 2022. | March 2021 – August 2022 | -Cultivate sponsors  -Secure and develop infrastructure resources and supports needed for the  program/ practice. | Process:  -Engagement (formally appointed internal implementation leaders) | -Identify and prepare champions  -Create new clinical teams  -Provide clinical supervision  -Distribute educational materials  -Facilitate relay of clinical data to providers  -Facilitation |
| Providers complete online trainings | Providers completed online asynchronous courses in OAAT therapy and SC 2.0. The online courses allowed staff to better understand the upcoming changes in the system and their role. | July 2021 – October 2021 | Provide initial training for practitioners | Inner Setting:  -Readiness for  Implementation (available resources, access to knowledge and information) | Conduct ongoing training |
| Offer live training in OAAT therapy with key field experts to providers | Providers completed a live, two-day training, with field experts in OAAT (Single-Session) therapy, to further their knowledge and abilities, and receive coaching | August 2021 –  October 2021 | Continue Training/Professional Learning as needed. | Inner Setting:  -Readiness for  Implementation (available resources, access to knowledge and information) | -Conduct ongoing training  -Make training dynamic  -Work with educational institutions |
| **Initial Implementation Stage** | | | | | |
| **NB Implementation Step** | **Description of NB Implementation Step** | **Timeframe** | **NIRN Components** | **CFIR Domains and Constructs** | **ERIC Strategies** |
| Implement OAAT therapy into addiction and mental health services | Providers started delivering OAAT therapy to clients on waitlists and expanded to offering OAAT therapy to new referrals and drop-in clients, as well as select clients on existing caseloads. | August 2021 – October 2021 | Practitioners begin delivery/use of program/practice. |  |  |
| Collect client feedback | Clients who received an OAAT therapy session were offered the opportunity to complete a client satisfaction survey at the end of the session.  This feedback was reviewed for improvement of service delivery. | October 2021 – March 2021 | Gather data and feedback through multiple sources including recipients and families to monitor progress. | Outer Setting:  -Patient Needs and  Resources  Process  -Engagement (clients | -Obtain and use feedback  from patients, consumers  and families |
| Utilize public awareness campaigns | A news release and press conference was delivered by Health Minister for launch of OAAT therapy. Further, OAAT therapy was added to the Bridgethegapp.ca website, which lists available Addiction and Mental Health services in NB. Program champions provided presentations and information to community stakeholders about the successes and value of OAAT therapy in CAMHCs. Some champions led awareness efforts, including engagement with family doctors, psychiatrists, new and existing clients, probation services, hospital social work, inpatient therapeutics and emergency services, social development contacts, First Nations community wellness teams, crown prosecutors, community inclusion networks, suicide prevention teams, primary care coordinators and other community care partners. | October 2021 – February 2022 |  | Inner Setting  -Networks and Communications | -Use mass media  -Conduct educational outreach visits |
| Complete post-implementation assessment | Post-implementation interviews were conducted by core project team with implementation leaders (e.g., local managers, clinical leads, and champions). The post-implementation assessment helped identify positive trends, outliers, and recommendations for improvement. | February 2022 | -Gather data and feedback through multiple sources including staff and practitioners to monitor progress.  -Use a process to develop improvement strategies through analysis of data and  feedback. | Process:  -Reflecting and Evaluating | -Purposely re-examine  the implementation.  -Facilitate relay of clinical data to providers |
| Improve implementation and delivery of OAAT therapy | By monitoring system-level data and feedback from providers and clients, the provincial working group assessed implementation and delivery of OAAT therapy and addressed challenges that arose. The feedback loop between the provincial working group (implementation team) and providers was vital in this process. | August 2021 – Present | -Use a process to develop improvement strategies through analysis of data and  feedback. | Process:  -Reflecting and Evaluating  Inner Setting:  -Implementation climate (learning climate, goals, and feedback) | -Purposely re-examine the implementation |
| **Full Implementation Stage** | | | | | |
| **NB Implementation Step** | **Description of NB Implementation Step** | **Timeframe** | **NIRN Components** | **CFIR Domains and Constructs** | **ERIC Strategies** |
| Train newly hired staff | To ensure sustainability, newly hired staff were trained with the online asynchronous courses and completed the two-day live OAAT therapy trainings with field experts. | October 2022 – Present | -Continue the training and coaching supports to maintain skillful use of the program or practice. |  | -Conduct ongoing training |
| Offer a community of practice | Clinical leads of CAMHCs began participating in a community of practice. Community of practice meetings foster continued professional development, allow for consultation with subject-matter experts, and provide a venue to highlight successes and address concerns. | May 2022 – Present | -Continue the training and coaching supports to maintain skillful use of the program or practice.  -Monitor and improve implementation supports and resources as needed to sustain implementation. | Process  -Executing | -Capture and share local knowledge  -Organize clinician implementation team meetings  -Create a learning collaborative |
| Supervisor Training | A supervisor training was held to help strengthen supervisory skills and develop more consistency in supporting providers delivering OAAT therapy. | September 2022 | -Monitor and improve implementation supports and resources as needed to  sustain implementation. | Intervention characteristics:  -Adaptability  Inner setting:  -Readiness for implementation (available resources, access to knowledge and information) | -Recruit, designate, and train for leadership  -Use train-the-trainer strategies |
| Implement fidelity measures for providers | A fidelity measure of OAAT therapy practices and adherence is presently being implemented. | November 2022 | -Continue to collect and use data for improvement (e.g., fidelity, program/process, outcome). | Process  -Executing | -Develop and implement tools for quality monitoring |
| Continue to review system data, operational guidelines, and stakeholder feedback | System data, operational guidelines, and stakeholder feedback will continue to be reviewed. | Ongoing | -Continue to collect and use data for improvement (e.g., fidelity, program/process, outcomes).  -Continue to use feedback loops with leadership, practitioners, staff, recipients and stakeholders to communicate about progress, improvement strategies and success. | Process:  -Reflecting and evaluating | -Obtain and use patients/consumers and family feedback |
